# Mitogen-activated protein kinase 14-mediated phosphorylation of MaMYB4 negatively regulates banana fruit ripening

**DOI:** 10.1093/hr/uhac243

**Published:** 2022-10-26

**Authors:** Yingying Yang, Chaojie Wu, Wei Shan, Wei Wei, Yating Zhao, Jianfei Kuang, Jianye Chen, Yueming Jiang, Wangjin Lu

**Affiliations:** State Key Laboratory for Conservation and Utilization of Subtropical Agro-bioresources/Guangdong Provincial Key Laboratory of Postharvest Science of Fruits and Vegetables/Engineering Research Center of Southern Horticultural Products Preservation, Ministry of Education, College of Horticulture, South China Agricultural University, Guangzhou 510642, China; State Key Laboratory for Conservation and Utilization of Subtropical Agro-bioresources/Guangdong Provincial Key Laboratory of Postharvest Science of Fruits and Vegetables/Engineering Research Center of Southern Horticultural Products Preservation, Ministry of Education, College of Horticulture, South China Agricultural University, Guangzhou 510642, China; State Key Laboratory for Conservation and Utilization of Subtropical Agro-bioresources/Guangdong Provincial Key Laboratory of Postharvest Science of Fruits and Vegetables/Engineering Research Center of Southern Horticultural Products Preservation, Ministry of Education, College of Horticulture, South China Agricultural University, Guangzhou 510642, China; State Key Laboratory for Conservation and Utilization of Subtropical Agro-bioresources/Guangdong Provincial Key Laboratory of Postharvest Science of Fruits and Vegetables/Engineering Research Center of Southern Horticultural Products Preservation, Ministry of Education, College of Horticulture, South China Agricultural University, Guangzhou 510642, China; State Key Laboratory for Conservation and Utilization of Subtropical Agro-bioresources/Guangdong Provincial Key Laboratory of Postharvest Science of Fruits and Vegetables/Engineering Research Center of Southern Horticultural Products Preservation, Ministry of Education, College of Horticulture, South China Agricultural University, Guangzhou 510642, China; State Key Laboratory for Conservation and Utilization of Subtropical Agro-bioresources/Guangdong Provincial Key Laboratory of Postharvest Science of Fruits and Vegetables/Engineering Research Center of Southern Horticultural Products Preservation, Ministry of Education, College of Horticulture, South China Agricultural University, Guangzhou 510642, China; State Key Laboratory for Conservation and Utilization of Subtropical Agro-bioresources/Guangdong Provincial Key Laboratory of Postharvest Science of Fruits and Vegetables/Engineering Research Center of Southern Horticultural Products Preservation, Ministry of Education, College of Horticulture, South China Agricultural University, Guangzhou 510642, China; South China Botanical Garden, Chinese Academy of Sciences, Guangzhou 510650, China; State Key Laboratory for Conservation and Utilization of Subtropical Agro-bioresources/Guangdong Provincial Key Laboratory of Postharvest Science of Fruits and Vegetables/Engineering Research Center of Southern Horticultural Products Preservation, Ministry of Education, College of Horticulture, South China Agricultural University, Guangzhou 510642, China

## Abstract

Mitogen-activated protein kinase (MAPK/MPK) cascades play crucial parts in plant growth, development processes, immune ability, and stress responses; however, the regulatory mechanism by which MAPK affects fruit ripening remains largely unexplored. Here, we reported that MaMPK14 cooperated with MaMYB4 to mediate postharvest banana fruit ripening. Transient overexpression of individual *MaMPK14* and *MaMYB4* in banana fruit delayed fruit ripening, confirming the negative roles in the ripening. The ripening negative regulator MaMYB4 could repress the transcription of genes associated with ethylene biosynthesis and fruit softening, such as *MaACS1*, *MaXTH5*, *MaPG3*, and *MaEXPA15*. Furthermore, MaMPK14 phosphorylated MaMYB4 at Ser160 via a direct interaction. Mutation at Ser160 of MaMYB4 reduced its interaction with MaMPK14 but did not affect its subcellular localization. Importantly, phosphorylation of MaMYB4 by MaMPK14 enhanced the MaMYB4-mediated transcriptional inhibition, binding strength, protein stability, and the repression of fruit ripening. Taken together, our results delineated the regulation pathway of MAPK module during banana fruit ripening, which involved the phosphorylation modification of MaMYB4 mediated by MaMPK14.

## Introduction

Fleshy fruit undergo dramatic changes during ripening, including variations in color, flavor, aroma, and texture [1]. Fruit ripening is a complex regulatory process consisting of dynamic interactions between multiple hormones, transcription factors (TFs), epigenetic and post-translational modifications, which ultimately leads to significant color change because of chlorophyll degradation and pigment accumulation, flavor improvement due to the synthesis of sugars, acids, and volatile compounds, as well as fruit softening as a result of cell wall remodeling [1, 2]. Thus, to elucidate the molecular genetic basis that underlies fruit ripening is an essential issue in postharvest biology.

Mitogen-activated protein kinase (MAPK/MPK) is one type of serine/threonine-type protein kinase. A typical MAPK cascade consists of three interconnected protein kinase modules (MAPKKK-MAPKK-MAPK) [3]. The MAPK cascade signaling pathway receives external signaling stimuli and transduces them into the cells through serial phosphorylation, which ultimately affect the expression of specific genes to convey the response [4]. In this process, downstream MAPK recognizes and acts on substrates consisting of cytoskeleton-associated proteins, TFs, or protein kinases [5]. The MAPK cascade has been reported to function in many signaling pathways in plants [4, 5]. For example, Arabidopsis MPK3/6 positively regulate the biosynthesis of camalexin, one of the major defense metabolites in plants, by elevating WRKY33 binding ability on the promoter of *CYP71B15* via phosphorylating WRKY33 [6]. Poplar LTF1 (a kind of MYB) which acts as a regulator of lignin biosynthesis is phosphorylated by PdMPK6 when external stimuli (e.g. trauma) occurs, leading to activation of lignin formation [7]. Additionally, the MAPK cascade OsMAPKK4-OsMAPK6 regulates brassinosteroid (BR) response by phosphorylating OsWRKY53 [8]. Similarly, banana MaMPK14 enhanced MaBZR1/2-mediated transcriptional repression of fruit ripening-related genes [9], while MaMPK6–3 phosphorylates MabZIP21, which in turn increases its transcriptional activation of ripening-associated genes [10]. Recently, rice OsMKKK70 participates in the OsMKK4-OsMAPK6-OsWRKY53 signaling pathway to regulate leaf angle and grain size [11]. *FaMAPK5* and *FaMAPK10* are related to ABA-mediated fruit ripening in strawberry [12]. These findings indicate that MAPK acts as an important player in fruit ripening, but more details remain largely unknown.

Over the past decade, multiple TFs have been revealed to engage in fruit ripening, including those necessary for ripening (e.g. NAC, MADS-box, and SBP-box), and others responsible for more subtle effects, such as ERF, bHLH, and WRKY [13–15]. Among these TFs, MYBs are reported to be extensively involved in various plant physiological procedures, such as growth and development, secondary metabolism, and response to stresses [16, 17]. MYB TFs such as TTG, CPC, WER, GL1, GL2, and GL3 from *Arabidopsis thaliana* are related to the formation of root hairs [18, 19], while AtMYB5/23 could regulate the extension and branching of epidermal hairs [16]. Members of the S6 subgroup (AtMYB75, AtMYB90, AtMYB113, etc.) are known to function in the regulation of anthocyanin metabolism [16]. In addition, MYBs in rice [20], potato [21], and maize [22] take part in the regulation of cell wall components, such as cellulose, lignin, and capsaicinoid. In most fruits, some members of MYB TFs play a role in regulation of pigment metabolism during fruit growth and development. Kiwifruit AdMYB7 regulated the accumulations of carotenoid and chlorophyll in fruit through the transcriptional activation of genes related to pigment metabolic pathways [23]. The SlMYB75 modulated the accumulation of anthocyanins which make tomato fruit purple [24]. In apple, MdMYB308L facilitated anthocyanin production by modulating the expression of anthocyanin biosynthetic genes, and overexpression or repression of *MdMYB308L* increased or decreased anthocyanin contents [25]. Interestingly, PtMYB4 is a substrate for PtMAPK6 in xylem development [26], and phosphorylation of AtMYB15 by AtMAPK6 is required for freezing tolerance [27], suggesting that MYB TFs could be regulated by MAPK; however, whether and how MAPK-MYB regulates fruit ripening regulation is unclear.

The MAPKs can be classified into four groups, in which the investigated best are MPK3/6 in group A and MPK4 in group B, while relatively little functional information has been reported about groups C and D [5, 28]. Here, we reported that MaMPK14 (Ma04_g03880), which belongs to the group C, is negatively involved in banana fruit ripening. Transient overexpression of *MaMPK14* delayed fruit ripening in banana. Furthermore, MaMYB4, one of the negative regulators in banana fruit ripening which could be degraded by MaBRG2/3 ubiquitination [29], was identified as a substrate of MaMPK14. Importantly, phosphorylation of Ser160 in MaMYB4 by MaMPK14 strengthened its transcriptional repression of fruit ripening-associated genes, DNA-binding strength, protein stability and the repression of fruit ripening. Overall, our work supports a model of MaMPK14-MaMYB4 module that negatively regulates banana fruit ripening.

## Results

### *MaMPK14* negatively regulates fruit ripening

**Figure 1:**
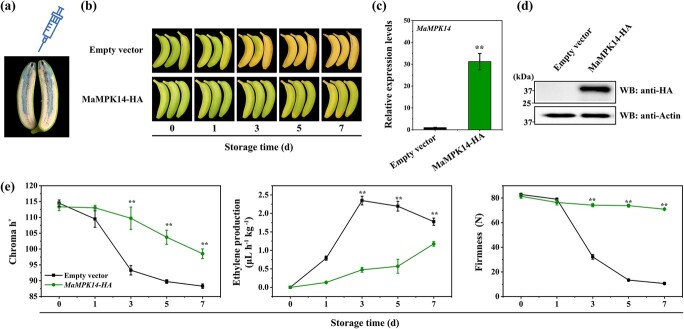
*MaMPK14* transient overexpression delays banana fruit ripening. **a** Illustration of successful injection of *Agrobacterium* solution into banana fruit. **b** The appearance of banana fruit during ripening was recorded for *MaMPK14* and control (empty vector) transiently overexpressing. **c** qRT-PCR and **d** Western blot determination of *MaMPK14* overexpression in banana fruit. **e** Patterns of variation in physiological indicators of infiltrated banana fruits.

The banana (*Musa acuminata* v1, 2012) genome possesses 25 MPKs [30], and we updated the gene ID (*M. acuminata* v2, 2016) through the banana genome database (https://bananagenome-hub.southgreen.fr/) (Table S1, see online supplementary material). Previously, we found that group C member MaMPK14 (Fig. S1, see online supplementary material) interacts with MaBZR1/2 to mediate banana fruit ripening [9], but the expression of *MaMPK14* and whether it regulates banana fruit ripening requires further elucidated. In this study, we analysed the expression of *MaMPK14* in natural ripening (control group), ethylene-induced ripening and 1-MCP-delayed ripening bananas (Figs S2 and S3, see online supplementary material) and analysed the related ripening characteristics in these different ripening bananas. As shown in Fig. S2 (see online supplementary material), ethylene treatment accelerated fruit ripening compared to the control, while negative effect was the case of 1-MCP. *MaMPK14* transcript was shown to decrease gradually during ripening, with minimal levels in late ripening stage (Fig. S3, see online supplementary material), following the same trend as for peel color and fruit hardness (Fig. S2b, see online supplementary material), consistent with previously reported data on banana transcriptomics [31]. To study more details of *MaMPK14* in fruit ripening, transient overexpression in banana fruit was performed (Fig. 1a–d). We found that transient overexpression of *MaMPK14* in banana fruit showed a significant delay of fruit ripening (Fig. 1b). Particularly, the peel of the expressed *MaMPK14* showed less yellowing compared to the control fruit (Fig. 1a and e). Likewise, the h° values which reflect the change in banana peel from green to yellow were higher in banana infiltrated with *MaMPK14* than that in the control (empty vector) fruit (Fig. 1e). Meanwhile, decrease of fruit firmness and ethylene production were suppressed in *MaMPK14-*overexpressed fruits (Fig. 1e). Collectively, *MaMPK14* plays a negative role in banana fruit ripening.

### MaMPK14 physically interacts with and phosphorylates MaMYB4

**Figure 2:**
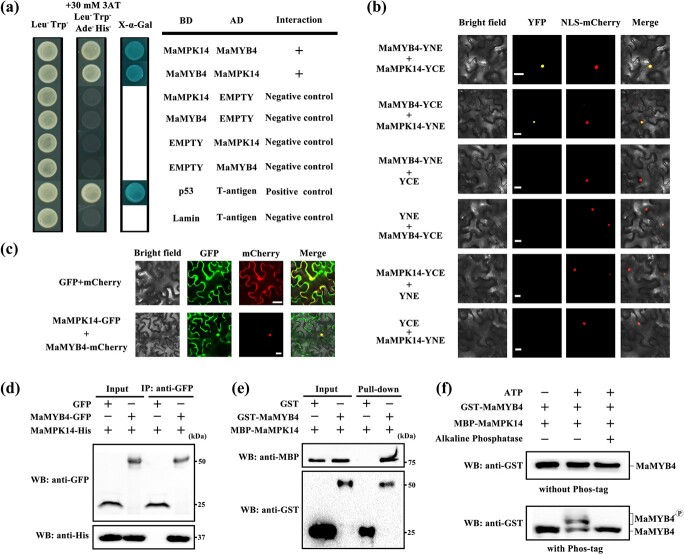
MaMYB4 is a substrate for MaMPK14. **a** Y2H assay for MaMPK14 and MaMYB4 interaction. The CDS of *MaMPK14* and *MaMYB4* were constructed into BD and AD vectors, respectively, then transferred into the yeast (Gold Y2H) strain. A positive result was recorded if the plates could be grown on SD/−Leu-Trp-Ade-His (containing 30 mM 3-AT) plates as well as turning blue in the presence of the chromogenic substrate X-α-Gal. **b** BiFC showing the interaction of MaMPK14 and MaMYB4. MaMPK14 and MaMYB4 was constructed onto YNE and YCE vector, respectively. The fusion constructs were introduced to tobacco leaves by agro-infiltration. Fluorescent signal was observed with fluorescence microscopy. Bars = 25 μm. **c** Co-localization of MaMPK14-GFP and MaMYB4-mCherry in *N. benthamiana*. Fluorescent signals of GFP and mCherry empty plasmids were applied as negative controls and NLS-mCherry was used as an indicator of nuclear localization. Bars = 25 μm. **d** MaMPK14 and MaMYB4 *in vivo* interaction was detected by CoIP. Tobacco leaves co-expressing His-MaMPK14 and MaMYB4-GFP or GFP, were used for immunoprecipitation with GFP antibody and performed western blot with His and GFP antibodies. **e** GST pull-down to investigate MaMPK14-MaMYB4 interactions. MBP-tag MaMPK14 protein was incubated with GST-tag MaMYB4 or GST protein, and then the bound protein was detected via western blotting with MBP and GST antibodies, respectively. **f** MaMPK14 phosphorylates MaMYB4 *in vitro.* GST-MaMYB4 protein was separated in Phos-tag SDS-PAGE gels and subjected to western blot with GST antibody. The upper band is indicated as phosphorylated MaMYB4 and the lower as unphosphorylated.

To explore the potential role of MaMPK14 in modulating fruit ripening, we used MaMPK14 as the bait to screen the banana fruit cDNA library by yeast two-hybrid (Y2H) screening. Since pGBKT7-MaMPK14 displayed self-activation, 3-amino-1,2,4-triazole (3-AT), a competitive inhibitor of His3 protein, was used for preventing its self-activation. By carefully screening, we obtained a positive clone MaMYB4 (Ma01_g19610) that interacted with MaMPK14 in yeast. To verify their interaction, we fused MaMPK14 and MaMYB4 to pGADT7 (AD) or pGBKT7 (BD) vectors, respectively. Further Y2H assays revealed that MaMPK14 actually interacted with MaMYB4 in yeast cells. As the negative control, MaMPK14 did not show interaction with the empty AD vector in Y2H assays (Fig. 2a). Then we used the BiFC assays to validate the interaction between MaMPK14 and MaMYB4. As shown in Fig. 2b, nuclear localization signals were detected in tobacco leaf cells co-expressing MaMYB4-YNE + MaMPK14-YCE and MaMPK14-YNE + MaMYB4-YCE vectors, while the negative controls (MaMYB4-YNE + YCE, YNE + MaMYB4-YCE, MaMPK14-YNE + YCE and YNE + MaMPK14-YCE) displayed no signal. A tobacco leaf transient expression system was used to verify the subcellular co-localization of MaMPK14 and MaMYB4 proteins. The results showed that MaMPK14 could co-localize with MaMYB4 in the nucleus (Fig. 2c). Moreover, *in vivo* CoIP assay was conducted by utilizing tobacco leaves transiently expressing MaMYB4-GFP and MaMPK14-His, with the combination of GFP and MaMPK14-His serving as a negative control. Fig. 2d showed that MaMPK14 could be immunoprecipitated by anti-GFP resin when co-expressed with MaMYB4-GFP. Consistently, *in vitro* GST pull-down assay employing recombinant MBP-MaMPK14 protein and GST-MaMYB4 protein were performed (Fig. S4, see online supplementary material,), whereas GST protein and recombinant MBP-MaMPK14 protein were used as negative controls. The MBP-tag MaMPK14 was pulled down by GST-tag MaMYB4 but not by GST control (Fig. 2e). These data demonstrated that MaMPK14 interacts with MaMYB4 both *in vivo* and *in vitro*. To study whether MaMPK14 phosphorylates MaMYB4, we carried out Phos-tag mobility shift assays. The MBP-MaMPK14 protein was incubated with GST-MaMYB4 protein and ATP presence. Immunoblot analysis showed that a slower mobility shift corresponding to the phosphorylated form of GST-MaMYB4 was observed in the incubation of MBP-MaMPK14, GST-MaMYB4, and ATP in Phos-tag SDS-PAGE gels (Fig. 2f). Altogether, these findings suggest that MaMPK14 interacts with and phosphorylates MaMYB4.

### Transient overexpression of *MaMPK14* in banana fruit substantially represses the expression of MaMYB4 and MaBZR1/2 target genes

**Figure 3:**
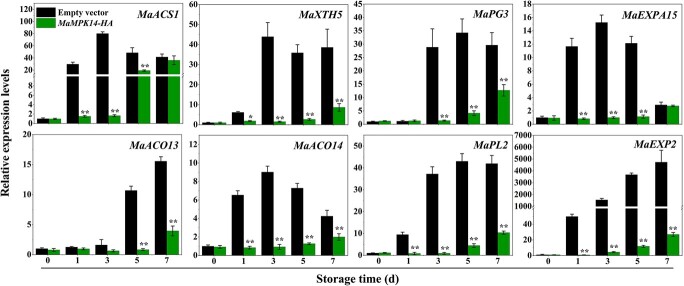
Relative expression of MaMYB4 and MaBRA1/2 target genes during transient overexpression of *MaMPK14* banana fruit ripening. Each gene’s expression level is expressed as a ratio relative to the 0 d of control group, it was set as 1. Each value represents the means ± SE of three replicates. The ^**^ and^ *^ denote significant differences between treatments (Student’s *t*-test, *P* < 0.05 or *P* < 0.01), respectively.

Our previous study indicated that MaMPK14 physically interacted with MaBZR1/2 [9]. To investigate the expression profiles of target genes of MaBZR1/2 (*MaACS1*, *MaACO13/14*, *MaEXP2*, *MaPL2*, and *MaXTH5*) as well as MaMYB4 (*MaACS1*, *MaXTH5*, *MaPG3*, and *MaEXPA15*) in *MaMPK14*-overexpressed banana fruit during ripening, qRT-PCR analyses were conducted. Results showed that expression of these ethylene synthesis-related genes (*MaACO13*, *MaACO14*, and *MaACS1*) and cell wall modification-related genes (*MaXTH5*, *MaPG3* and *MaEXPA15*, *MaEXP2*, and *MaPL2*) were significantly repressed in banana fruits overexpressing *MaMPK14* (Fig. 3), implying that MaMPK14 may affect the expression of these ripening-related genes by interacting with the MaBZR1/2 and MaMYB4 TFs.

### S160A mutation in MaMYB4 impairs its interaction with MaMPK14 but does not affect its subcellular localization

**Figure 4:**
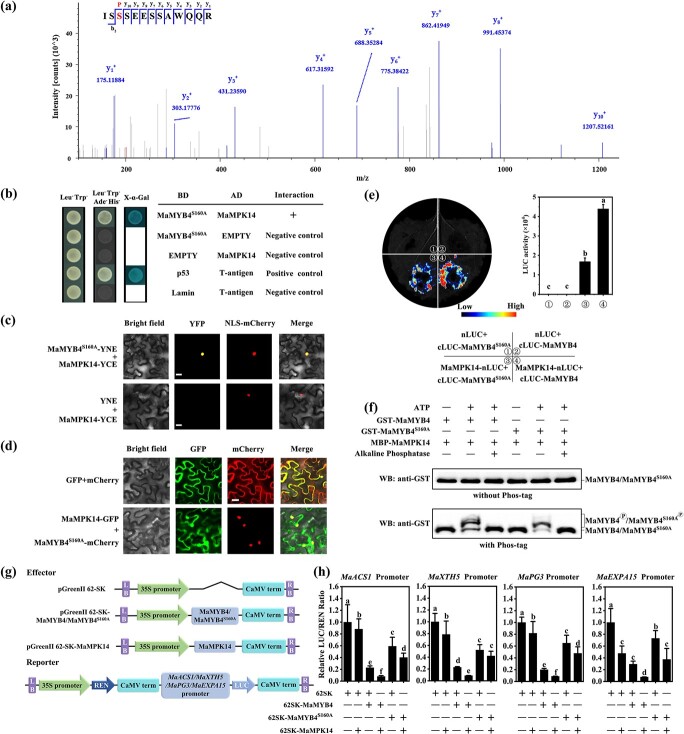
Substitution of Ser160 with Ala160 weakened the phosphorylation and transcriptional repression activity of MaMYB4. **a** Mass spectrometry analysis showed that Ser-160 in MaMYB4 was phosphorylated by MaMPK14 *in vivo*. **b** The interaction of MaMYB4^S160A^ with MaMPK14 was investigated by Y2H assay. The CDS of MaMPK14 and MaMYB4^S160A^ were constructed into BD and AD vectors, respectively, then transferred into the yeast (Gold Y2H) strain. Positive result was recorded if the plates could be grown on SD/−Leu-Trp-Ade-His plates as well as turning blue in the presence of the chromogenic substrate X-α-Gal. **c** BiFC assay of MaMPK14 and MaMYB4^S160A^ interaction. MaMYB4^S160A^ was constructed onto YNE vector and MaMPK14 constructed onto YCE vector. They were infiltrated to tobacco leaves and observed with fluorescence microscopy. Bar = 25 μm. **d** Co-localization of MaMPK14-GFP and MaMYB4^S160A^-mCherry in *N. benthamiana*. Fluorescent signals of GFP and mCherry empty plasmids were applied as negative controls, and NLS-mCherry served as an indicator for nuclear localization. Bar = 25 μm. **e** Firefly luciferase complementation imaging assay was performed to compare the strength of interaction between MaMYB4 or MaMYB4^S160A^ and MaMPK14 in *N. benthamiana* leaves. Then Luc/MaMYB4-cLuc, MaMPK14-nLuc/MaMYB4-cLuc and nLuc/MaMYB4^S160A^-cLuc, MaMPK14-nLuc/MaMYB4^S160A^-cLuc were co-transformed into tobacco leaves and examined. Each value represents the average SE of six biological replicates. Statistical comparison of means was performed via one-way ANOVA at the *P* < 0.05 level. **f***In vitro* kinase assay comparing MaMYB4 and MaMYB4^S160A^ phosphorylation by MaMPK14. MBP-MaMPK14 protein was incubated with GST-MaMYB4 or GST-MaMYB4^S160A^ of ALP and ATP in the presence (+) or absence (−) in buffer. GST-MaMYB4 or GST-MaMYB4^S160A^ proteins were separated in Phos-tag SDS-PAGE gels and performed western blot with GST antibody. **g** Schematic diagram of vector construction. **h** Effects of MaMYB4^S160A^ on the transcription of *MaACS1*, *MaXTH5*, *MaPG3*, and *MaEXPA15*. Substitution of Ser160 with Ala attenuated MaMYB4 transcriptional repression activity. Each value represents the average SE of six biological replicates. Statistical comparison of means was performed via one-way ANOVA at the *P* < 0.05 level.

To further investigate the possible phosphorylation sites of MaMYB4 by MaMPK14, we transiently co-expressed MaMYB4-GFP and MaMPK14-His in tobacco leaves and immunoprecipitated MaMYB4 protein using GFP antibody. A Ser160 phosphorylation site was recognized in MaMYB4 after LC–MS/MS analysis (Fig. 4a; Table S2, see online supplementary material). To ascertain the function of this phosphorylation site, we substituted Ser160 with Ala160 (MaMYB4^S160A^) to mimic the non-phosphorylation form of MaMYB4 (Fig. 4a). To investigate whether MaMYB4^S160A^ affects the interaction between MaMYB4 and MaMPK14, we performed Y2H, BiFC, subcellular co-localization, and LCI assays. As shown in Fig. 4b–e, MaMYB4^S160A^ still physically interacted with MaMPK14, but the interaction was substantially weakened (Fig. 4e). Additionally, the phosphorylation of MaMYB4 and MaMYB4^S160A^ was detected using the Phos-tag assays. As shown in Fig. 4f, the shifted band that denotes phosphoprotein was obviously detected in MaMYB4 in the presence of ATP. By contrast, relatively lower content of shifted band was observed in that of MaMYB4^S160A^. Furthermore, the phosphorylation of MaMYB4 and MaMYB4^S160A^ could be reduced after alkaline phosphatase treatment. These results suggest that MaMPK14 phosphorylates MaMYB4 mainly at Ser160.

### MaMPK14-mediated phosphorylation of MaMYB4 enhances its transcriptional repressive ability

Our previous study demonstrated that MaMYB4 was a transcriptional repressor [32]. To investigate the effect of phosphorylation of MaMYB4 by MaMPK14 on the transactivation activity of MaMYB4, DLR assay was performed (Fig. 4g–h). The *MaMYB4* expression alone repressed its target genes transcription, as indicated by a lower relative LUC/REN ratio than that of the empty vector (control). Interestingly, overexpression of *MaMPK14* alone also repressed the activities of *MaACS1*, *MaXTH5*, *MaPG3*, and *MaEXPA15*, implying that MaMPK14 might affect gene transcription through interaction of other uncharacterized mechanism. Notably, additionally repressive effects on the transcription of *MaACS1*, *MaXTH5*, *MaPG3*, and *MaEXPA15* were observed when *MaMPK14* and *MaMYB4* were co-expressed, indicating that MaMPK14 enhances MaMYB4-mediated transcriptional repression of ripening-associated genes. However, no further repressive effects of *MaACS1*, *MaXTH5*, *MaPG3*, and *MaEXPA15* were detected when *MaMYB4^S160A^* was transiently expressed (Fig. 4h). Its repression was significantly restored when *MaMPK14* was co-expressed, apart from *MaXTH5*, indicating that MaMPK14-mediated phosphorylation of Ser160 in MaMYB4 was essential for MaMYB4 transcriptional repression.

### Phosphorylation by MaMPK14 elevates MaMYB4’s DNA-binding capacity and boosts its stability

**Figure 5:**
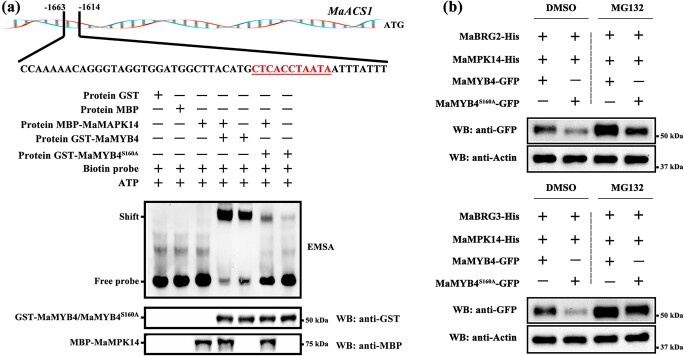
MaMPK14-mediated phosphorylation of MaMYB4 enhances its DNA-binding ability and stability. **a** Phosphorylation of MaMYB4 by MaMPK14 enhances its DNA-binding ability. The phosphorylated form of MaMYB4 enhanced ability to bind DNA, whereas the dephosphorylated version reduced its DNA-binding capacity. Labelled probe was incubated with phosphorylated MaMYB4 or MaMYB4^S160A^ by MaMPK14 and unphosphorylated MaMYB4 or MaMYB4^S160A^ in a control reaction without MaMPK14. Protein inputs were detected using an GST or MBP antibody. **b** GFP-tagged MaMYB4 with or without S/A substitution at S160 was transiently co-expressed with His-tagged MaMPK14 and His-tagged MaBRG2/3 (as E3, interacting with and ubiquitinating MaMYB4) in *N. benthamiana* leaves by agroinfiltration. Protein accumulation was examined by western blotting at 3 d after infiltration. MG132 was applied 12 h before sample harvesting, DMSO was used as a mock control, and Actin served as a loading control.

**Figure 6:**
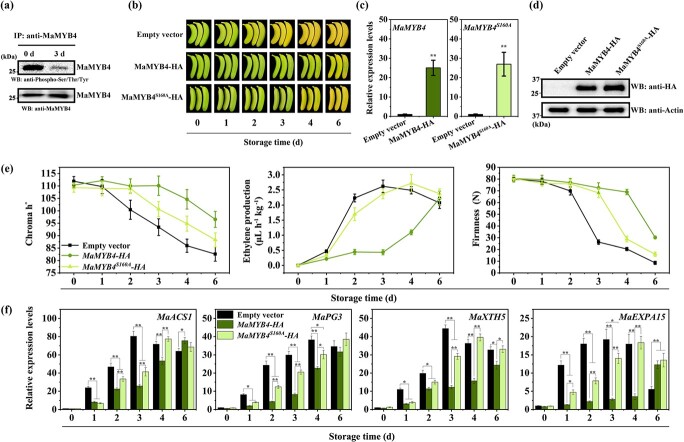
Phosphorylation of MaMYB4 represses fruit ripening. **a** Phosphorylation abundance of MaMYB4 in unripe and ripe banana fruit, respectively. The MaMYB4 proteins were immunoprecipitated using anti-MaMYB4 antibody from the proteins extracted in unripe and ripe banana at 0 and 3 d after ethylene treatment, and then subjected to immunoblot analysis using anti-phospho-Ser/Thr/Tyr and anti-MaMYB4 served as a loading control. **b** Photograph of banana fruit during ripening was recorded for *MaMYB4*, *MaMYB4^S160A^,* and control (empty vector) transiently overexpressing. **c** qRT-PCR and **d** Western blot to determine overexpression of *MaMYB4* or *MaMYB4^S160A^* overexpression in banana fruit. **e** Changes in physiological indicators of infiltrated banana fruits at each storage time. **f** Relative mRNA abundance of *MaACS1*, *MaXTH5*, *MaPG3*, and *MaEXPA15* in *MaMYB4* or *MaMYB4^S160A^* overexpressed and control fruits during fruit ripening. Each gene’s expression level is expressed as a ratio relative to the 0 d of control group, it was set as 1. Each value represents the means ± SE of three to six biological replicates. The ** and * denote significant differences between treatments (Student’s *t*-test, *P* < 0.05 or *P* < 0.01), respectively.

The DNA-binding strength of phosphorylated MaMYB4 was investigated using the EMSA assay. As shown in Fig. 5a, the DNA-binding capacity of MaMYB4 to the promoter of target gene was enhanced following the addition of purified MBP-MaMPK14, as compared to GST-MaMYB4 alone. In contrast, regardless of the incubation of MBP-MaMPK14 or not, the non-phosphorylatable mutant MaMYB4^S160A^ exhibited less DNA-binding strength. The negative controls including GST, MBP, or MBP-MaMPK14 protein alone displayed no binding bands. These observations suggest that MaMPK14-mediated phosphorylation of MaMYB4 increases its DNA-binding capacity. Since MaMYB4 has been shown to be degraded by E3 ubiquitin ligase MaBRG2/3 [29], we performed transient transformation assays in tobacco leaves (Fig. 5b) to further demonstrate whether MaMYB4 phosphorylation affects its stability. When *His-MaBRG2/3*, *His-MaMPK14*, and *MaMYB4^S160A^-GFP* were co-transferred into tobacco leaves, the stability of MaMYB4^S160A^ protein was greatly diminished compared with co-transferring *His-MaBRG2/3*, *His-MaMPK14*, and *MaMYB4-GFP*. In addition, when we treated with 100 μM MG132, an inhibitor of the 26S proteasome, the levels of MaMYB4 and MaMYB4^S160A^ increased greatly (Fig. 6b). These findings indicate that MaMYB4 is degraded by the 26S proteasome pathway and that MaMPK14-mediated MaMYB4 phosphorylation may prevent its degradation by MaBRG2/3.

### Phosphorylation of MaMYB4 represses fruit ripening

MaMYB4 was immunoprecipitated from total proteins in unripe and ripe banana fruits using anti-MaMYB4 antibody, and then followed by phosphorylation detection using antiphospho-Ser/Thr/Tyr antibody. The phosphorylation level of MaMYB4 was much higher in unripe banana than in ripe fruit (Fig. 6a). Furthermore, we transiently overexpressed *MaMYB4* and *MaMYB4^S160A^* in banana fruit, respectively, and investigated their regulatory effect on fruit ripening (Fig. 6b–d). Similar with the results observed in transient overexpression of *MaMPK14*, expression of *MaMYB4* also substantially delayed fruit ripening, with slower change from green to yellow in the peel of *MaMYB4*-transgenic fruit than control fruit. Also, decrease in fruit firmness and increase in ethylene production were suppressed in *MaMYB4*-transgenic fruit (Fig. 6e). Meanwhile, accumulation of *MaACS1*, *MaXTH5*, *MaPG3*, and *MaEXPA15* was significantly down-regulated during ripening (Fig. 6f). Interestingly, as shown in Fig. 6e–f, transient expression of *MaMYB4^S160A^* prolonged ripening, but the effect was significantly weaker than that of MaMYB4, indicating that phosphorylation of MaMYB4 enhanced its repression of fruit ripening. Consistently, the change of ripening parameters and ripening-associated gene expression showed similar trends (Fig. 6e–f).

## Discussion

Since the first discovery of MAPK in *Medicago sativa*, numerous MAPKs have been identified in various plants, including 20 MAPKs in Arabidopsis [33], 17 members in rice [34], and 25 members in banana [30]. MAPK is the last component in the triple mitogen-activated kinase signaling cascade, which plays a role in many biological procedures such as plant growth and development, apoptosis, and stress adaptation [4]. For example, AtMPK3 in Arabidopsis might be activated by environmental and oxidative stresses [35]. AtMPK9 was predominantly accumulated in guard cells, with a positively regulation by ROS in the ABA signaling pathway [36]. AtMPK18 could function in microtubules of plant cell cortex [37]. In rice, the activity of OsMAPK44 was induced by drought, salt, and oxidative stresses but not by cold stress [38], while OsMAPK33 activity was elevated under drought stress but decreased under salt stress [39]. Interestingly, several studies have shown that MAPKs are related to the regulation of fruit ripening. Previously, our research found that MaMPK2 may affect banana ripening through phosphorylation of MabZIP93 resulting in transcriptional activation of on cell wall-modifying genes [40]. Another MAPK in banana MaMAPK11–3 cooperated with MabZIP74 to modulate *MaACO1/4* transcription during fruit ripening [41]. More recently, MaMPK6–3 interacted with and phosphorylated MabZIP21 to promote banana fruit ripening [10].

In this study, we found that the expression of *MaMPK14* was downregulated during fruit ripening (Fig. S3, see online supplementary material) and overexpression of *MaMPK14* delayed banana fruit ripening (Fig. 1), demonstrating that MaMPK14 is a negative regulator of fruit ripening. However, the exact mechanisms that underlie the involvement of MaMPK14 in banana ripening remain largely unclear. Our prior study showed that MaMPK14 interacted with MaBZR1/2, suggesting that MaBZR1/2 are potential substrates of MaMPK14. In this context, using Y2H, BiFC, co-localization, CoIP, and pull-down technologies, MaMYB4 was identified as another interaction partner of MaMPK14 (Fig. 2). Furthermore, the expressions of *MaACO13*, *MaACO14*, *MaACS1*, *MaXTH5*, *MaPG3*, *MaEXPA15*, *MaEXP2*, and *MaPL2*, the target genes of MaMYB4 and MaBZR1/2, were inhibited in *MaMPK14*-overexpression banana fruit (Fig. 3), implying that MaMPK14 may negatively regulate banana fruit ripening through association with MaBZR1/2 and MaMYB4. A previous report has shown that MaMYB4 is a transcriptional repressor localizing in the nucleus [32]. In this context, we found that the expression of *MaMYB4* is inhibited by ethylene (Fig. S3, see online supplementary material). It seems presumable that the inhibition of *MaMYB4* by ethylene treatment eliminated its repressive effect on ripening-associated genes *MaACS1*, *MaXTH5*, *MaPG3*, and *MaEXPA15*, which in turn leads to ethylene-induced fruit softening. Given that MaMPK14 is a member of MAPK (Fig. S1, see online supplementary material) and the phosphorylation content of MaMYB4 is diminished during banana ripening (Fig. 6a), we speculated that MaMYB4 was a substrate of MaMPK14, as in the cases of MYB15 [27], LTF1 [7], and MYB75/PAP1 [42]. To validate our speculation, phos-tag assays were used in this investigation. We found that MaMPK14 was able to phosphorylate MaMYB4 *in vitro* (Fig. 2f), indicating that MaMYB4 was modulated at the post-translational level. Transient overexpression of *MaMYB4* in banana not only delayed fruit ripening, but also downregulated the expression of ethylene biosynthetic gene *MaACS1* and cell wall modified genes *MaXTH5*, *MaPG3*, and *MaEXPA15* (Fig. 6). Altogether, these observations provide evidence that MaMPK14-mediated MaMYB4 phosphorylation is involved in banana fruit ripening.

Protein phosphorylation might affect protein interaction, subcellular localization, DNA-binding capacity, and stability [27]. For example, phosphorylated LTF1 as a sensitive switch regulates lignin biosynthesis in plant response to environmental stimuli [7]. MPK4-mediated phosphorylation of MYB75 increased its stability to regulate light-induced anthocyanin accumulation [42]. In addition, phosphorylation of MYB42 by MPK4 enhanced its protein stability and transcriptional activity under salt stress conditions [43]. In this study, we found that Ser160 of MaMYB4 was an important residue phosphorylated by MaMPK14 (Fig. 4). MaMYB4^S160A^ did not alter its subcellular localization (Fig. 4d) but reduced both interaction with MaMPK14 (Fig. 4b–e) and overall phosphorylation contents (Fig. 4f). It should be pointed out that except for Ser160, additional phosphorylation sites may exist in MaMYB4, as mutation of Ser160 in MaMYB4 still displayed phosphorylation (Fig. 4f). Unfortunately, we failed to obtain new phosphorylation sites, possibly due to the complex structure of MaMYB4 or the relatively weak phosphorylation signal that is difficult to detect. Furthermore, protein phosphorylation strengthened MaMYB4-mediated transcriptional inhibition of the target genes, but this inhibitory effect was reduced in the non-phosphorylation mutant MaMYB4^S160A^ (Fig. 4h). Our results were similar to the findings of Zhang *et al.* [44], who showed that AabZIP1^S37A^ also physically interacts with AaAPK1. Protein phosphorylation has an effect on TF-mediated transcriptional activity. To gain insight into the molecular mechanism by which MaMPK14 affects MaMYB4’s function, we performed dual-luciferase reporter assays. We found that transcriptions of *MaACS1*, *MaXTH5*, *MaPG3*, and *MaEXPA15* were significantly repressed when *MaMYB4* was transiently expressed, while the repression was further strengthened when *MaMPK14* and *MaMYB4* were co-expressed (Fig. 4f), suggesting that the interaction between MaMYB4 and MaMPK14 enhanced MaMYB4-mediated transcriptional inhibition on a subset of ripening-associated genes: *MaACS1*, *MaXTH5*, *MaPG3*, and *MaEXPA15*. As reported by Shan *et al.* [9], co-expression of MaBZR1/2 with MaMPK14 further repressed the transcription of the target genes. On the contrary, co-expression of MaMAPK11–3 and MabZIP74 diminished the transcriptional repressive ability of MabZIP74 [41]. Furthermore, the phosphorylation of MaMYB4 by MaMPK14 enhanced its DNA-binding ability and protein stability (Fig. 5). Similarly, ZmMPK5 phosphorylation of ZmNAC49 strengthened its binding ability but had no effect on its transactivation activity [3]. MPK4-mediated phosphorylation of MYB75 increased its stability to regulate light-induced anthocyanin accumulation [42]. Besides, phosphorylation of MYB42 by MPK4 enhanced its protein stability and transcriptional activity under salt stress conditions [43]. On the contrary, WRKY3 is destabilized by phosphorylation SnRK1 *in planta* [45]. Notably, transient expression of *MaMYB4^S160A^* resulted in a delayed ripening phenotype, and the effect was intermediate between those expressing *MaMYB4-HA* and the empty vector *pCXUN-HA* (Fig. 6b–f), suggesting that phosphorylation of MaMYB4 by MaMPK14 enhances its inhibition of fruit ripening. With the rapid development of new technological tools such as CRISPR/Cas9 and other gene editing techniques in banana fruit [46], it would be interesting to apply these technologies to validate further the gene functions of MaMYB4 and MaMPK14 in banana fruit ripening.

**Figure 7:**
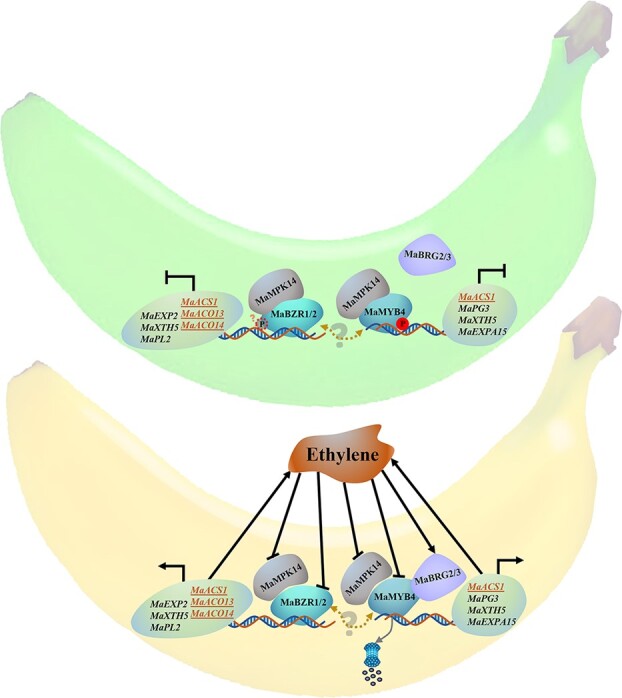
Proposed model of MaMPK14 in controlling banana ripening. As for the MaMPK14-MaMYB4 module, in unripe banana, highly expressed MaMPK14 phosphorylates a transcriptional repressor MaMYB4 mainly at Ser160 site to form a repressor complex that synergistically represses the transcription of ripening-associated genes *MaACS1*, *MaPG3*, *MaXTH5*, and *MaEXPA15*, which represses their transcriptional expression. During fruit ripening, accumulation of *MaMPK14* and *MaMYB4* is inhibited by ethylene and accumulation of *MaBRG2/3* is induced. MaBRG2/3 ubiquitinate MaMYB4 for proteasome-dependent degradation, which weakens the repressive effect on the ripening-associated genes, resulting in gene activation and fruit ripening. As for the MaMPK14-MaBZR1/2 module, a similar regulatory mechanism may exist in which MaMPK14 negatively regulates banana fruit ripening by interacting with MaBZR1/2 and affecting their transcriptional activity on the target genes (*MaACS1*, *MaACO13*, *MaACO14*, *MaPL2*, *MaXTH5*, and *MaEXP2*). A solid line indicates the confirmed findings, whereas a dotted line implies the predicted results. Arrows denote positive regulation, while a T bar represents negative regulation.

In summary, we proposed a regulatory model (Fig. 7), in which MaMPK14 negatively regulates banana fruit ripening through phosphorylation of MaMYB4 or MaBZR1/2, thus affecting the expression of ripening-associated genes. In unripe banana, highly expressed MaMPK14 phosphorylates a transcriptional repressor MaMYB4 mainly at Ser160 site to form a repressor complex that synergistically represses the transcription of ripening-associated genes. Meanwhile, phosphorylation of MaMYB4 by MaMPK14 prevents MaMYB4 degradation caused by MaBRG2/3. During fruit ripening, accumulation of *MaMPK14* and *MaMYB4* is inhibited by ethylene and accumulation of *MaBRG2/3* is induced. MaBRG2/3 ubiquitinate MaMYB4 for proteasome-dependent degradation, which weakens the repressive effect on the ripening-associated genes, leading to gene activation and thus ethylene production and fruit softening. As for the MaMPK14-MaBZR1/2 module, there are still many links to be refined, such as whether MaMPK14 phosphorylates MaBZR1/2 to affect the transcriptional activity of MaBZR1/2. In addition, whether MaMYB4 interacts with MaBZR1/2 also needs to be further investigated in future direction. Our findings shed light on the regulatory mechanism underlying postharvest banana fruit ripening at the post-translational level, which can provide gene resource for banana improvement through genetic engineering strategy.

## Materials and methods

### Materials, plant growth conditions, and treatments

The 70–80% mature stage of banana fruit (*M. acuminata* L., group AAA) were hand-harvested from an orchard near Guangzhou, China. Three treatments were used based on our prior study [15], including a control group (only storage at 22°C), ethylene-induced ripening group (100 μL L^−1^ ethylene treatment for 16 h followed by storage at 22°C), and 1-MCP-delayed ripening group (0.5 μL L^−1^ 1-MCP treatment for 16 h followed by storage at 22°C). During storage, samples were collected according to the ripening progression which was evaluated by ripening parameters. All samples were rapidly frozen via immersion in liquid nitrogen and stored at −80°C pending analyses. Each treatment had three replicas.

Tobacco (*Nicotiana benthamiana*) plants were cultivated at 22°C under long daylight conditions (8 h of darkness/16 h of light) in a growth chamber and selected from 4 to 6 weeks old for transient expression experiments.

### Phylogenetic relationship analysis and amino acid sequence comparison

Phylogenetic analysis was constructed using MEGA-X with a bootstrap test of phylogeny following the neighbor-joining method. Multiple sequence comparison analysis was conducted using CLUSTALW and Jalview software.

### Dual-luciferase reporter (DLR) transient expression assay

The transcriptional effects of MaMYB4, MaMYB4^S160A^ and MaMPK14 on *MaACS1*, *MaPG3*, *MaXTH5*, *MaPG3*, and *MaEXPA15* transcription were measured using a DLR transient expression system in tobacco leaves, as mentioned previously [13]. Target gene promoters were individually cloned as reporter into the pGreenII 0800-LUC vector as reporters, whereas MaMYB4, MaMYB4^S160A^, and MaMPK14 were ligated as effectors into the pGreenII 62-SK vector as effectors. Transiently co-expressed different effector and reporter plasmid pairs in tobacco leaves. Detection of lucifierase activity was described above. The activities of LUC and REN were quantified on a Thermo Scientific Luminoskan Ascent apparatus using the Dual Luciferase Reporter Gene Assay Kit (Yeasen, Shanghai, China). Each pair includes at least six biological replicates.

### Analysis of banana pulp transient overexpression

The coding sequences (CDSs) of *MaMPK14*, *MaMYB4*, and *MaMYB4^S160A^* were individually cloned into pCXUN-HA vector, which were controlled by the maize (*Zea mays*) Ubiquitin promoter [47]. The recombinant MaMYB4-HA, MaMPK14-HA constructs and the empty vector pCXUN-HA were transferred to strain EHA105. The 2 mL of suspensions comprising MaMPK14-HA, MaMYB4-HA, MaMYB4^S160A-^HA or control pCXUN-HA were then injected into green banana fruit by distal injection, respectively, at 22°C, incubated for 24 h, as previously described [15]. The infiltrated fruit were then treated with 100 μL L^−1^ ethylene at 16 h and stored at 22°C for 7 days with 90% relative humidity. Samples were collected for measurement of fruit color, ethylene production, pulp firmness, and gene expression, and each assay was referred to our previous report [15].

### Y2H assay

The CDSs of *MaMPK14* was constructed into pGBKT7 as bait, operated according to the handbook of Y2H screening kit [9]. A selection medium was supplemented with 3-AT to exclude the autonomous activation of pGBKT7-MaMPK14. To confirm the interaction between MaMPK14 and MaMYB4/MaMYB4^S160A^, they were constructed into pGBKT7 (BD) or pGADT7 (AD) vectors as bait and prey, respectively, as per the instructions. Possible interactions were assessed based on plates could be grown on SD/−Leu-Trp-Ade-His (containing 3-AT) plates as well turn blue in the presence of the chromogenic substrate X-α-Gal.

### BiFC assay

The CDSs of *MaMYB4*, *MaMYB4^S160A^*, and *MaMPK14* were constructed into pUC-pSPYNE or pUC-pSPYCE vectors, respectively [48]. Expression of *MaMYB4*, *MaMYB4^S160A^*, and *MaMPK14* alone was used as negative controls, while NLS-mCherry fluorescence signal was used as an indicator for the nucleus, which was then manipulated as mentioned previously [13]. Fluorescence microscope (Zeiss Axioskop 2 plus, Carl Zeiss Microscopy GmbH, Munich, Germany) was used to detect the fluorescent signals of YFP and mCherry.

### Co-localization assay

The CDSs of *MaMYB4*, *MaMYB4^S160A^,* and *MaMPK14* were cloned into pEAQ-HT-mCherry and pEAQ-HT-GFP vectors, respectively. The GFP vector, mCherry vector and fusion plasmid were electroporated into *Agrobacterium* strain EHA105, followed by transient expression in tobacco leaf as described previously [49]. The empty GFP and mCherry vectors were used as negative controls. Fluorescent photographs of GFP and mCherry were taken at 36–48 h after infiltration.

### Protein expression, purification, and GST pull-down assay

The CDSs of *MaMPK14*, *MaMYB4*, and *MaMYB4^S160A^* were introduced into pMAL-c2X and pGEX-4 T-1vector, respectively, then transformed into *Escherichia coli* strain BM Rosetta (DE3). They were induced with isopropyl β-D-1-thiogalactopyranoside (IPTG) to express GST-MaMYB4/GST- MaMYB4^S160A^ or MBP-MaMPK14 as previously reported [32]. The above GST and MBP recombinant proteins were purified with a MBP (New England Biolabs, Beverly, MA, USA) or Pierce™ GST (Yeasen, Shanghai, China) spin purification kits, respectively.

GST pull-down assay was conducted by reference to the Cold Spring Harbor Protocols with minor modifications [50]. Briefly, GST and GST-MaMYB4, MBP-MaMPK14 protein were measured by western blot using the GST antibody (Thermo Fisher Scientific, Waltham, MA, USA) and MBP antibody (Sigma). Horse-radish peroxidase was detected using the chemiluminescent substrate SuperSignal West Pico (Bio-Rad, Hercules, CA, USA) and imaged on the ChemiDoc™ MP Imaging Apparatus (Bio-Rad, Hercules, CA, USA).

### CoIP assay, LC–MS/MS analysis, and protein stability assay

The CDSs of *MaMPK14* and *MaMYB4* were constructed into pEAQ-HT (with His-tag) and pEAQ-HT-GFP vectors, transfused into *Agrobacterium tumefaciens* strain EHA105 and injected into tobacco, respectively. CoIP assay were undertaken as described previously [32]. Samples were detected by immunoblotting with GFP and His antibody (Abcam, Cambridge, MA, USA), respectively.

For *in vivo* phosphorylation analysis, tobacco leaves injected with MaMYB4-GFP + MaMPK14-His and MaMYB4-GFP + His fusion proteins were subjected to a mixture containing sodium pyrophosphate, 20 mM Tris (pH 7.5), 150 mM NaCl, 1% Triton X-100, β-glycerophosphate, EDTA, Na_3_VO_4_, and leupeptin (Beyotime, Haimen, China) in IP buffer for lysis. Enrichment of MaMYB4-GFP protein was performed using anti-GFP (Abcam, Cambridge, MA, USA). After immunoprecipitation, the samples were separated in 10% SDS-PAGE gels, and the protein bands corresponding to MaMYB4-GFP were sectioned and analysed using LC–MS/MS at Jingjie PTM BioLab (Hangzhou) Co. Ltd.

For protein stability assay, MaMYB4-GFP with or without S/A substitution at S160 was transiently co-expressed with MaMPK14-His and MaBRG2/3-His [29] (as E3, interacting with and ubiquitinating MaMYB4) in *N. benthamiana* leaves, followed by transient expression in tobacco leaf as described previously [29]. Protein accumulation was examined by western blotting at 3 d after infiltration. MG132 was applied 12 h before sample harvesting, DMSO was used as a mock control. Samples were detected by immunoblotting with GFP (Abcam, Cambridge, MA, USA) and Actin (Abbkine, Shanghai, China) antibody, respectively.

### Firefly luciferase complementation imaging (LCI) assay

The CDSs of *MaMYB4*, *MaMYB4^S160A^*, and *MaMPK14* were constructed into the pCAMBIA1300-Cluc and pCAMBIA1300-Nluc vectors, respectively [51]. The above fusion plasmids were inserted into strain GV3101 (P19) and injected into tobacco leaves. Luminescence of luciferase activity was measured 36–48 h later using Luciferase Reporter Gene Assay Kit (Yeasen, Shanghai, China). Luciferase activity is referred to the section on DLR assay, and the luminescence images were captured on a CCD imaging system (Bio-Rad, Hercules, CA, USA).

### Phosphorylation assay *in vitro*

Phosphorylation assay *in vitro* was conducted as described previously [40, 44]. Briefly, 1 μg of MBP-MaMPK14 and 3 μg of GST-MaMYB4 proteins were added with a total volume of 25 μL of reaction buffer containing 20 mM Tris–HCl, 20 mM MgCl_2_, 100 mM NaCl, 10 mM ATP, 2 mM DTT. For combinations with alkaline phosphatase (ALP), 1 μL of ALP (Thermo Fisher Scientific, Waltham, MA, USA) was mixed into 25 μL of incubation solution. Each sample was gently mixed and incubated at 30°C for 25 min, then separated by 10% (*w*/*v*) SDS-PAGE with 0.1 mM MnCl_2_ and 0.1 mM Phos-tag (Wako Pure Chemical, Osaka, Japan), followed by detection of the proteins with GST antibody (Thermo Fisher Scientific, Waltham, MA, USA).

### Electrophoresis mobility shift assay (EMSA)

Expression and purification of GST-MaMYB4, GST-MaMYB4^S160A^, and MBP-MaMPK14 proteins as described are detailed in the section on GST pull-down assays. Biotin labeling of the DNA fragments (50 bp) containing YYYACCWAMYW [29] (Text S1, see online supplementary material) in the promoter of *MaACS1* and EMSA reaction, referring to the kit mentioned in Fan *et al.* [13]. To test the effect of MaMPK14-mediated phosphorylation on the DNA-binding activity of MaMYB4, recombinant MBP-MaMPK14 was mixed with GST-MaMYB4 or GST-MaMYB4^S160A^ protein in reaction buffer containing 20 mM Tris–HCl buffer, 20 mM MgCl_2_, 100 mM NaCl, 10 mM ATP, 2 mM DTT, for 0.5 h at 30°C before performing the EMSA.

### Data analysis

A Student’s *t*-test (*P* < 0.05 or *P* < 0.01) was used to determined statistical differences between two samples, and a one-way ANOVA at the *P* < 0.05 level was used for statistical comparisons of multiple samples.

### Primer sequences

A list of all primers used in the present study is given in Table S3 (see online supplementary material).

## Acknowledgments

This work was funded by the National Natural Science Foundation of China (Grant No. 31830071) and China Agriculture Research System of Ministry of Finance (MOF) and Ministry of Agriculture and Rural affairs (MARA) (Grant No. CARS-31).

## Author contributions

W.L. conceived and designed the experiments. Y.Y. performed most of the experiments and analysed the data. C.W., W.W., and Y. Z. performed some of the experiments. Y.Y. wrote the manuscript. J.K. revised the manuscript. W.S., J.C., and Y. J. gave advice for the manuscript. All the authors have read and approved the final version of the manuscript.

## Data availability

All relevant data can be found within the manuscript and its supporting materials.

## Conflict of interest

The authors declare no conflicts of interest.

## Supplementary data

Supplementary data is available at *Horticulture Research* online.

## Supplementary Material

Web_Material_uhac243
