# Supplementary material for: Mitogen-activated protein kinase 14-mediated phosphorylation of MaMYB4 negatively regulates banana fruit ripening
Source: Hortic Res. 2022 Oct 26;10(1):uhac243. doi: 10.1093/hr/uhac243 (PMC9832833; doi:10.1093/hr/uhac243)
Supplement: Web_Material_uhac243 [file web_material_uhac243.docx]

**Supplementary information**

**
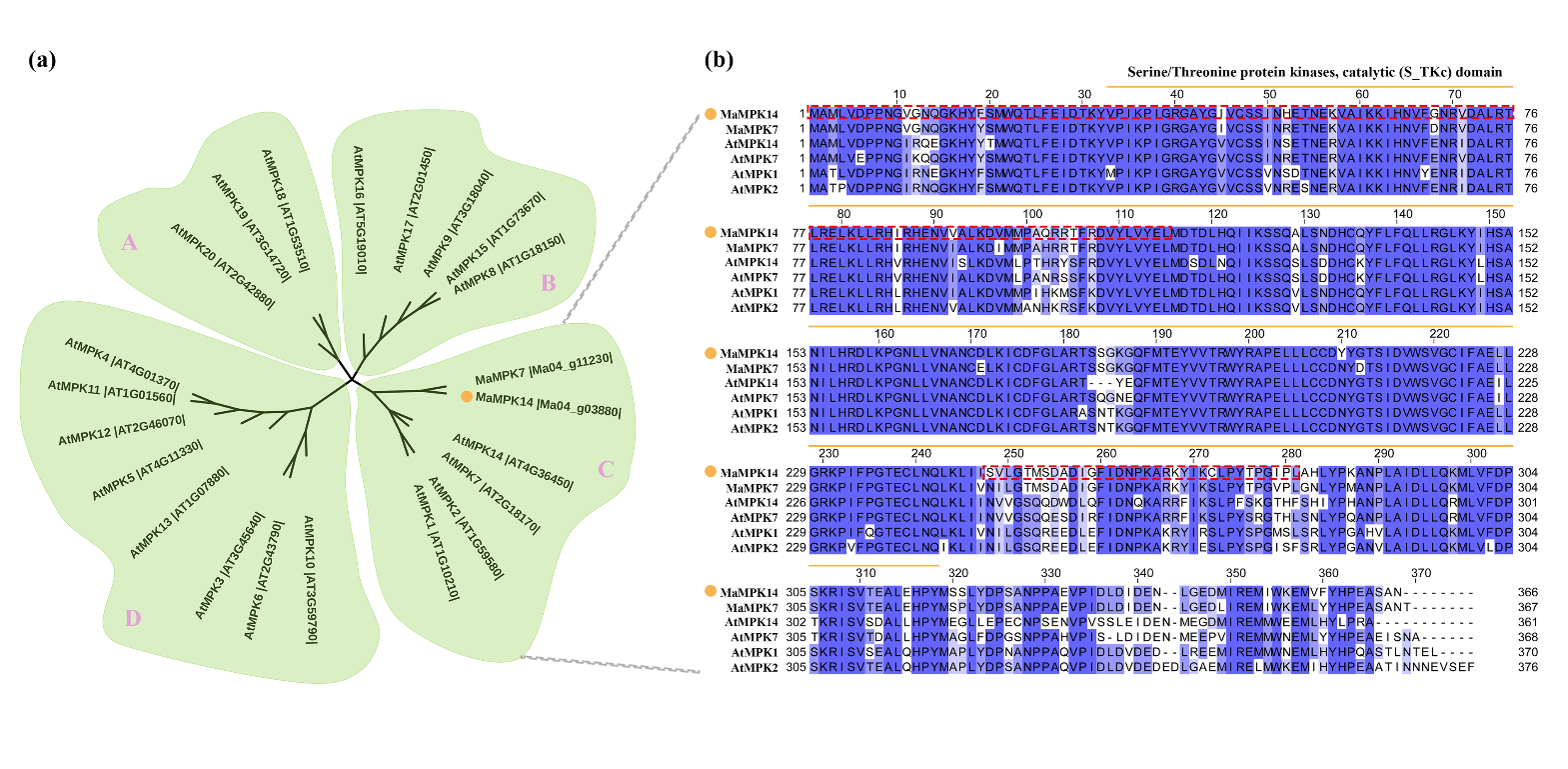
**

**Fig. S1. Phylogenetic and multiple sequence alignment analysis of MaMPK14 (Ma04_g03880) protein sequence. a** Phylogenetic analysis of MaMPK14 with the MAPK proteins from Arabidopsis. In banana, MaMPK14 and MaMPK7 were classified in group C. The phylogenetic tree was constructed using MEGA-Ⅹ with a bootstrap test of phylogeny following the neighbor-joining method. Bootstrapping with 1,000 replicates was used to assess the statistical reliability of nodes in the tree. **b** Multiple sequence comparison analysis of group CMAPK proteins in Arabidopsis and banana using CLUSTALW and Jalview software. The red dashed box indicates the extra amino acids in the MaMPK14 (Ma04_g03880) protein sequence over GSMUA_Achr4G03760_001^29^. The orange line indicates Serine/Threonine protein kinases, catalytic (S_TKc) domain

**
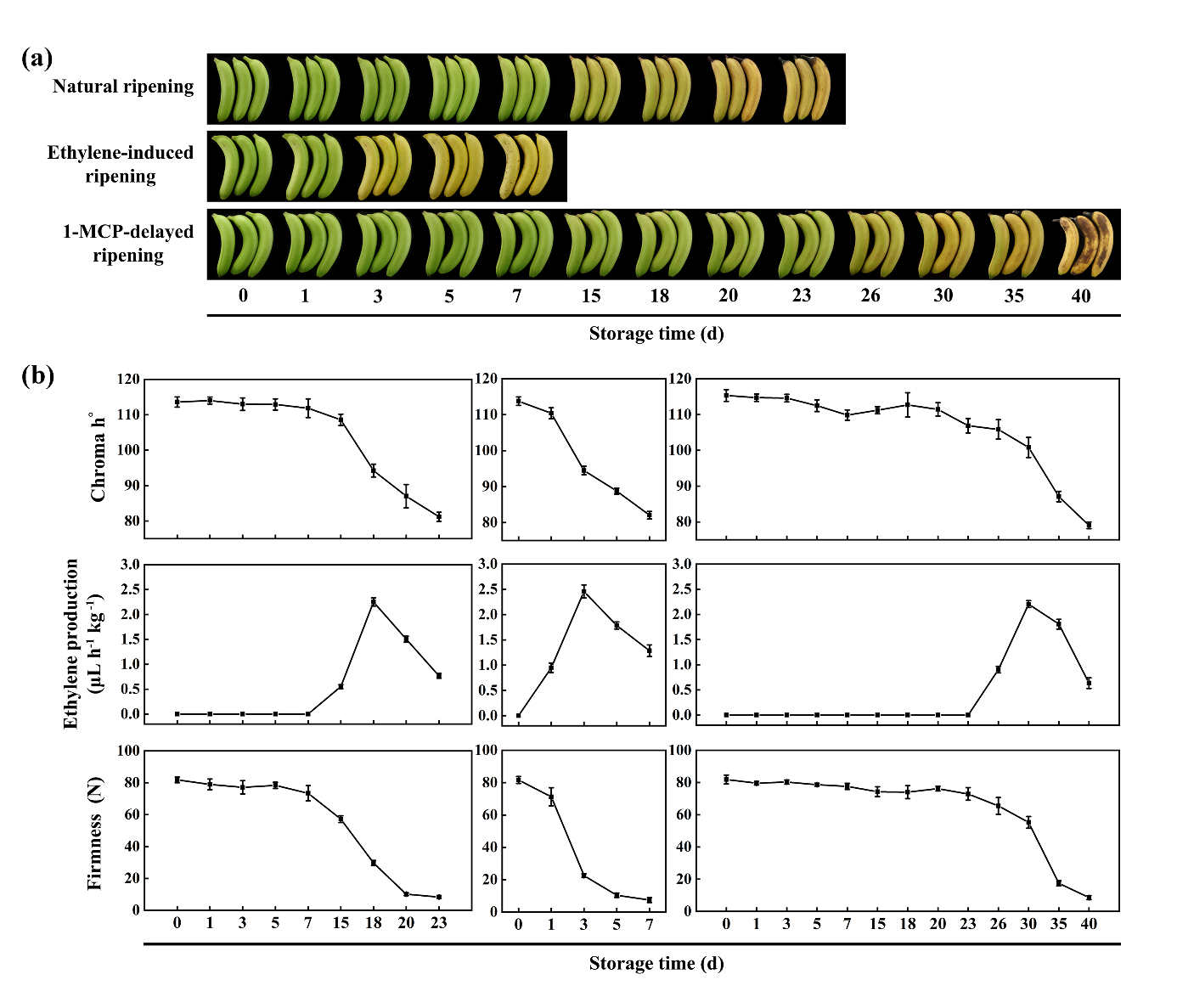
**

**Fig.** **S2.** **Phenotypic and physiological changes in banana fruit ripening. a** Three postharvest treatments were performed, including natural, ethylene-induced and 1-MCP-delayed ripening groups (banana fruit stored at 22°C or 100 μL L^−1^ ethylene/0.5 μL L^−1^ 1-MCP, 16 h for 16 h at 22°C). **b** Changes of peel chroma, ethylene production and fruit firmness in three groups. Each value represents the means ± SE of three replicates

**
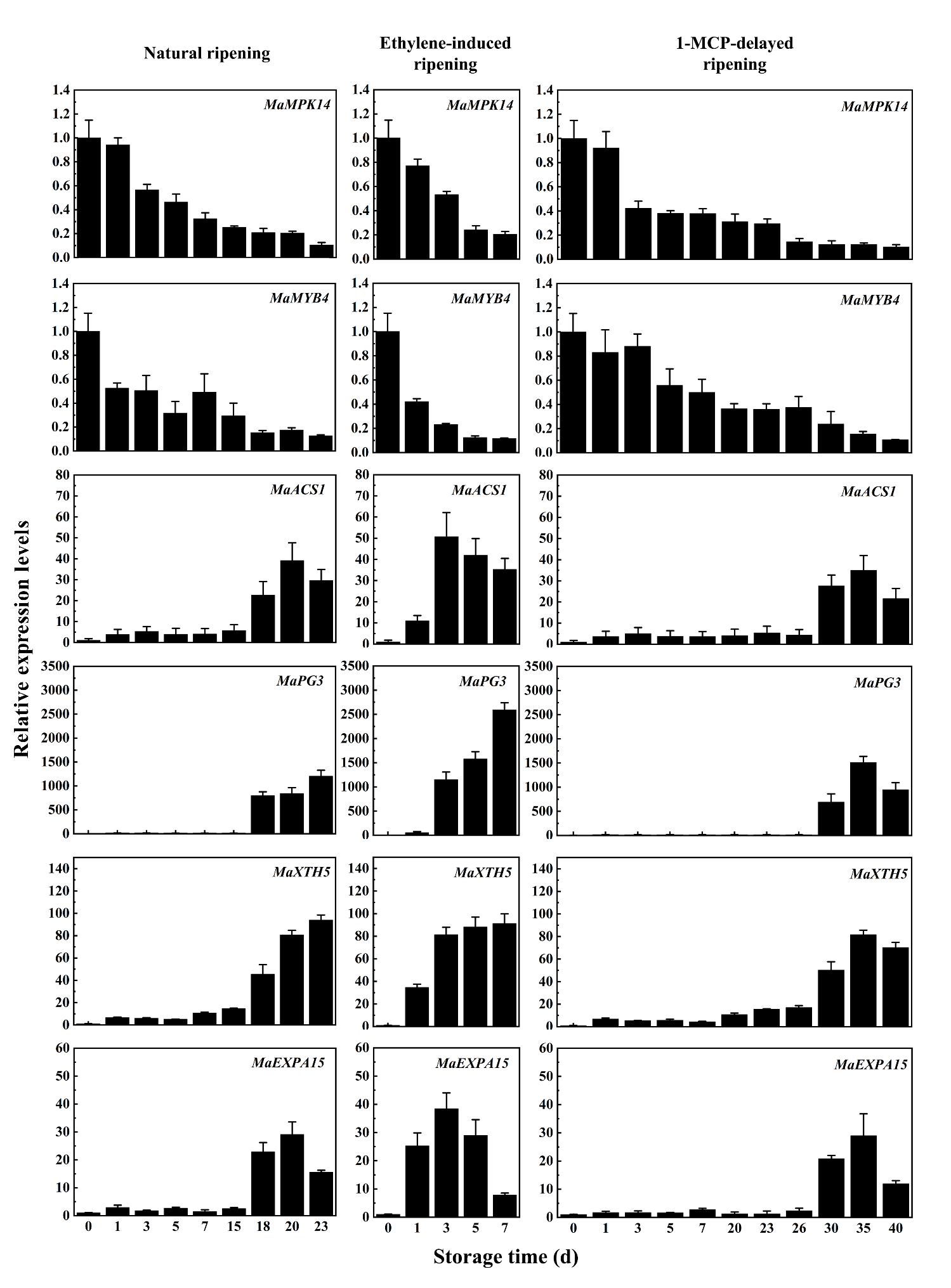
**

**Fig. S3.** **Expression of *MaMPK14*, *MaMYB4*, *MaACS1*, *MaPG3*, *MaXTH5* and *MaEXPA15* in fruit ripening.** The expression levels of each gene are expressed as a ratio relative to the harvest time (0 d of control), which was set as 1. Each value represents the mean ± S.E. of three replicates

**
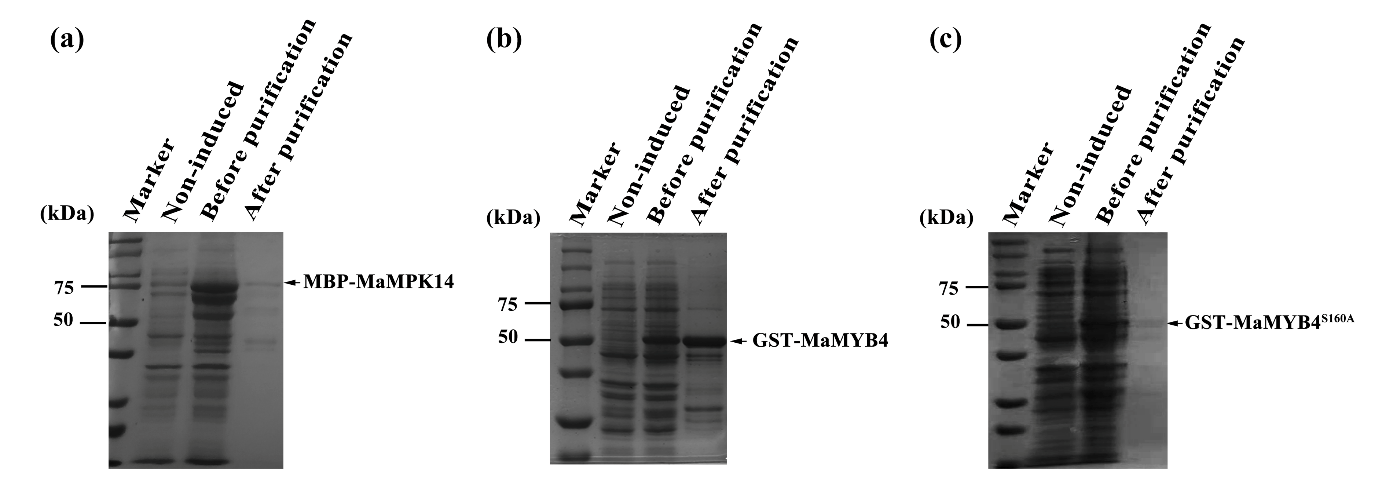
**

**Fig. S4.** SDS-PAGE gel demonstrating purification of the MBP-tag MaMPK14, GST-tag MaMYB4 and GST-tag MaMYB4^S160A^ protein used for study


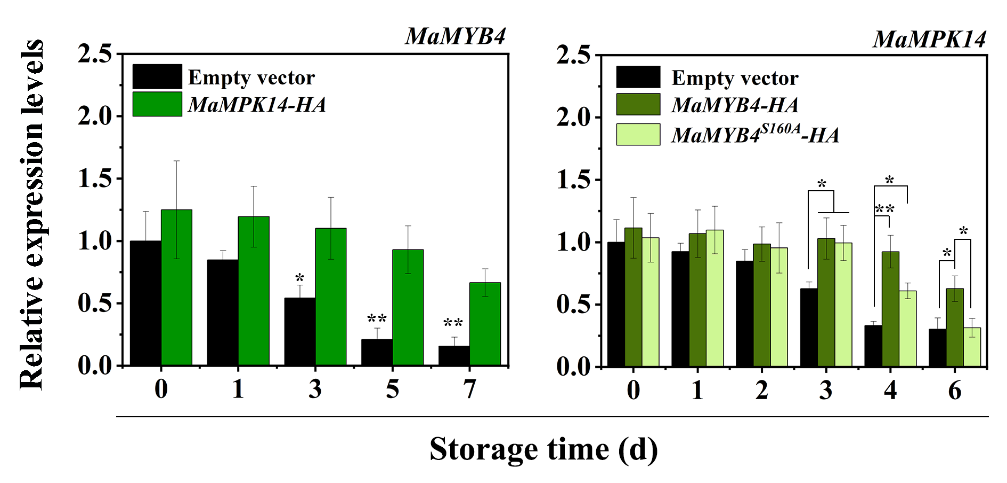


**Fig. S5.** Relative expression of *MaMYB4* during transient overexpression of *MaMPK14* banana fruit ripening and relative expression of *MaMPK14* during transient overexpression of *MaMYB4* or *MaMYB4^S160A^* banana fruit ripening. Each gene’s expression level is expressed as a ratio relative to the 0 d of control group, it was set as 1. Each value represents the means ± SE of three to six biological replicates. The ** and * denote significant differences between treatments (Student’s *t*-test, *P*<0.05 or *P*<0.01), respectively

**Table S1.** Nomenclature for MAPKs in *Musa acuminata*.

| Name | Query ID | Reference | Gene code validation in the banana genome database | |
| --- | --- | --- | --- | --- |
|  |  |  | **V2 (2016)** | **V1 (2012)** |
| MaMPK3-1 | GSMUA_Achr6G33100_001 | Asif et al., 2014 | Ma06_g34790 | GSMUA_Achr6G33100_001 |
| MaMPK3-2 | GSMUA_Achr9G04280_001 | Asif et al., 2014 | Ma09_g04230 | GSMUA_Achr9G04280_001 |
| MaMPK4 | GSMUA_Achr11G02910_001 | Asif et al., 2014 | Ma11_g02790 | GSMUA_Achr11G02910_001 |
| MaMPK7 | GSMUA_Achr4G11270_001 | Asif et al., 2014 | Ma04_g11230 | GSMUA_Achr4G11270_001 |
| MaMPK6-1 | GSMUA_Achr10G00980_001 | Asif et al., 2014 | Ma10_g06650 | GSMUA_Achr10G00980_001 |
| MaMPK6-2 | GSMUA_Achr5G06640_001 | Asif et al., 2014 | Ma05_g06410 | GSMUA_Achr5G06640_001 |
| MaMPK6-3 | GSMUA_Achr2G19320_001 | Asif et al., 2014 | Ma02_g21170 | GSMUA_Achr2G19320_001 |
| MaMPK9-1 | GSMUA_Achr1G20760_001 | Asif et al., 2014 | Ma01_g14080 | GSMUA_Achr1G20760_001 |
| MaMPK9-2 | GSMUA_Achr9G00750_001 | Asif et al., 2014 | Ma09_g00730 | GSMUA_Achr9G00750_001 |
| MaMPK9-3 | GSMUA_Achr4G03750_001 | Asif et al., 2014 | Ma04_g03870 | GSMUA_Achr4G03750_001 |
| MaMPK9-4 | GSMUA_Achr4G11240_001 | Asif et al., 2014 | Ma04_g11200 | GSMUA_Achr4G11240_001 |
| MaMPK11-1 | GSMUA_Achr4G04470_001 | Asif et al., 2014 | Ma04_g04500 | GSMUA_Achr4G04470_001 |
| MaMPK11-2 | GSMUA_Achr4G11650_001 | Asif et al., 2014 | Ma04_g11610,  Ma04_g11620,  Ma04_g11630 | GSMUA_Achr4G11630_001, GSMUA_Achr4G11640_001, GSMUA_Achr4G11650_001 |
| MaMPK11-3 | GSMUA_Achr4G05570_001 | Asif et al., 2014 | Ma04_g05600 | GSMUA_Achr4G05570_001 |
| MaMPK11-4 | GSMUA_Achr4G12170_001 | Asif et al., 2014 | Ma04_g12170 | GSMUA_Achr4G12170_001 |
| MaMPK14 | GSMUA_Achr4G03760_001 | Asif et al., 2014 | Ma04_g03880 | GSMUA_Achr4G03760_001,  GSMUA_Achr4G03770_001 |
| MaMPK20-1 | GSMUA_Achr9G06770_001 | Asif et al., 2014 | Ma09_g06560 | GSMUA_Achr9G06770_001, GSMUA_Achr9G06780_001 |
| MaMPK20-2 | GSMUA_Achr6G27100_001 | Asif et al., 2014 | Ma06_g28840 | GSMUA_Achr6G27100_001 |
| MaMPK20-3 | GSMUA_AchrUn_randomG17900_001 | Asif et al., 2014 | Ma00_g00010 | GSMUA_AchrUn_randomG17900_001, GSMUA_AchrUn_randomG17910_001 |
| MaMPK20-4 | GSMUA_Achr3G03110_001 | Asif et al., 2014 | Ma03_g02940 | GSMUA_Achr3G03110_001 |
| MaMPKX-1 | GSMUA_Achr9G22050_001 | Asif et al., 2014 | Ma09_g22590 | GSMUA_Achr9T22050_001, GSMUA_Achr9G22050_001 |
| MaMPKX-2 | GSMUA_Achr9G22040_001 | Asif et al., 2014 | Ma09_g22580 | GSMUA_Achr9T22040_001, GSMUA_Achr9G22040_001 |
| MaMPKX-3 | GSMUA_Achr11G02860_001 | Asif et al., 2014 | Ma11_g02770,  Ma11_g02780 | GSMUA_Achr11G02850_001, GSMUA_Achr11G02860_001, GSMUA_Achr11G02870_001, GSMUA_Achr11G02880_001, GSMUA_Achr11G02890_001, GSMUA_Achr11G02900_001 |

**Table S2.** List of LC-MS ion identifications.

| **#1** | **b⁺** | **b²⁺** | **Seq.** | **y⁺** | **y²⁺** | **z⁺** | **z²⁺** | **#2** |
| --- | --- | --- | --- | --- | --- | --- | --- | --- |
| 1 | 114.09135 | 57.54931 | I |  |  |  |  | 13 |
| 2 | 201.12338 | 101.06533 | S | 1461.56424 | 731.28576 | 1445.54552 | 723.27640 | 12 |
| 3 | 368.12174 | 184.56451 | S-Phospho | 1374.53221 | 687.76974 | 1358.51349 | 679.76038 | 11 |
| 4 | 455.15377 | 228.08052 | S | 1207.53385 | 604.27056 | 1191.51513 | 596.26120 | 10 |
| 5 | 584.19637 | 292.60182 | E | 1120.50182 | 560.75455 | 1104.48310 | 552.74519 | 9 |
| 6 | 713.23897 | 357.12312 | E | 991.45922 | 496.23325 | 975.44050 | 488.22389 | 8 |
| 7 | 800.27100 | 400.63914 | S | 862.41662 | 431.71195 | 846.39790 | 423.70259 | 7 |
| 8 | 887.30303 | 444.15515 | S | 775.38459 | 388.19593 | 759.36587 | 380.18657 | 6 |
| 9 | 958.34015 | 479.67371 | A | 688.35256 | 344.67992 | 672.33384 | 336.67056 | 5 |
| 10 | 1144.41947 | 572.71337 | W | 617.31544 | 309.16136 | 601.29672 | 301.15200 | 4 |
| 11 | 1272.47805 | 636.74266 | Q | 431.23612 | 216.12170 | 415.21740 | 208.11234 | 3 |
| 12 | 1400.53663 | 700.77195 | Q | 303.17754 | 152.09241 | 287.15882 | 144.08305 | 2 |
| 13 |  |  | R | 175.11896 | 88.06312 | 159.10024 | 80.05376 | 1 |

**Table S3.** Summary of primers used in this study.

| **Assay** | **Primer sequence (5’-3’)** | | **Restriction site** |
| --- | --- | --- | --- |
| **RT-qPCR** | ***MaRPS4-qF: TGAGAGTGGCTTGACCCTGA***  ***MaMYB4-qF: TCAAGAACTACTGGAACACCC***  ***MaMPK14-qF: GTTGCTGCGTGGATTGAAGTAT***  ***MaACS1-qF: ACAAGTTCAAGATCACCCAAGC***  ***MaXTH5-qF: CGCGAGAGGTACATGATCTACT***  ***MaPG3-qF: CTGCTGTGTGTTGATGATCGT***  ***MaEXPA15-qF: CAGCATTTCGATCTCTCTCAGC***  ***MaACO13-qF: GAACAAAGGAAACCGCATCTTC***  ***MaACO14-qF: GAATGGGGTTTCTTTCAGCTGG***  ***MaEXP2-qF: TATGGAGGGGGTGATGCTT***  ***MaPL2-qF: GGCTCCACTGCCATTACG*** | ***MaRPS4-qR: GTGACATTTAGTCGTCTGCTGG***  ***MaMYB4-qR: CTGTTGGGAAGGAGGGCTTATA***  ***MaMPK14-qR: ATGCATCCAACTGACCAAACAT***  ***Ma ACS1-qR: AGTGCATCCTTTTCTCGTTGAC***  ***MaXTH5-qR: CTACTCCGATGACTGAACGTTG***  ***MaPG3-qR: CGATCACATGGTATACACGCG***  ***MaEXPA15-qR: GAACCTGATCCCTCCTCTCTTC***  ***MaACO13-qR: TGGTCCCGAAGTAGTAATGGAG***  ***MaACO14-qR: CTCCTCCCTCAACTTGTAGCA***  ***MaEXP2-qR: CGTGCTCAGTGCCGTGTTC***  ***MaPL2-qR: GTGCGTGTAGTCATTGTTTACC*** |  |
| **Full length cloning** | ***MaMYB4/MaMYB4^S160A^-F: ATGGGGAGGTCCCCGTG***  ***MaMYB4/MaMYB4^S160A^-R: CTATGGGTTTCTGTAGTCCAGCA***  ***MaMPK14-F: ATGGCTATGCTGGTTGATCCTC***  ***MaMPK14-R: TCAGTTAGCAGAAGCTTCTGGATGATA*** | |  |
| **Y2H assay** | ***MaMYB4/MaMYB4^S160A^-AD-F: ggaggccagtgaattcATGGGGAGGTCCCCGTG***  ***MaMYB4/MaMYB4^S160A^-AD-R: cgagctcgatggatccCTATGGGTTTCTGTAGTCCAGCA***  ***MaMPK14-AD-F: ggaggccagtgaattcATGGCTATGCTGGTTGATCCTC***  ***MaMPK14-AD-R: cgagctcgatggatccTCAGTTAGCAGAAGCTTCTGGATGATA***  ***MaMYB4/MaMYB4^S160A^-BD-F: catggaggccgaattcATGGGGAGGTCCCCGTG***  ***MaMYB4MaMYB4^S160A^-BD-R: gccgctgcaggtcgacgCTATGGGTTTCTGTAGTCCAGCA***  ***MaMPK14-BD-F: catggaggccgaattcATGGCTATGCTGGTTGATCCTC***  ***MaMPK14-BD-R: gccgctgcaggtcgacgTCAGTTAGCAGAAGCTTCTGGATGATA*** | | ***Eco*R Ⅰ**  ***Bam*H I**  ***Eco*R Ⅰ**  ***Bam*H I**  ***Eco*R Ⅰ**  ***Sal* Ⅰ**  ***Eco*R Ⅰ**  ***Sal* Ⅰ** |
| **Subcellular localization and Co-localization** | ***MaMYB4/MaMYB4^S160A^-GFP-F: tattctgcccaaattcgcgaccggtATGGGGAGGTCCCCGTG***  ***MaMYB4/MaMYB4^S160A^-GFP-R: aaagttcttctcctttgctagtcatTGGGTTTCTGTAGTCCAGCA***  ***MaMPK14-GFP-F: tattctgcccaaattcgcgaccggtATGGCTATGCTGGTTGATCCTC***  ***MaMPK14-GFP-R: aaagttcttctcctttgctagtcatGTTAGCAGAAGCTTCTGGATGATA***  ***MaMYB4/MaMYB4^S160A^-mCherry-F: ttctgcccaaattcgcgaccggtATGGCAGTCGAAACCCACCATC***  ***MaMYB4/MaMYB4^S160A^-mCherry-R: tcctcctcgcccttgctcaccatAGACATGTTCACAATGACAGTCC*** | | ***Age* Ⅰ**  ***Age* Ⅰ**  ***Age* Ⅰ**  ***Age* Ⅰ**  ***Age* Ⅰ**  ***Age* Ⅰ** |
| **BiFC assay** | ***MaMYB4/MaMYB4^S160A^-YNE-F: ggcgcgccactagtggatccATGGGGAGGTCCCCGTG***  ***MaMYB4/MaMYB4^S160A^-YNE-R:*** ***gcggtaccctcgaggtcgacTGGGTTTCTGTAGTCCAGCA***  ***MaMPK14-YNE-F: ggcgcgccactagtggatccATGGCTATGCTGGTTGATCCTC***  ***MaMPK14-YNE-R: gcggtaccctcgaggtcgacGTTAGCAGAAGCTTCTGGATGATA***  ***MaMYB4/MaMYB4^S160A^-YCE-F: ggcgcgccactagtggatccATGGGGAGGTCCCCGTG***  ***MaMYB4/MaMYB4^S160A^-YCE-R:*** ***gcggtaccctcgaggtcgacTGGGTTTCTGTAGTCCAGCA***  ***MaMPK14-YCE-F: ggcgcgccactagtggatccATGGCTATGCTGGTTGATCCTC***  ***MaMPK14-YCE-R: gcggtaccctcgaggtcgacGTTAGCAGAAGCTTCTGGATGATA*** | | ***Bam*H I**  ***Sal* I**  ***Bam*H I**  ***Sal* I**  ***Bam*H I**  ***Sal* I**  ***Bam*H I**  ***Sal* I** |
| **Promoter islation** | ***MaMYB4-pro-F: CTCTGTTAATATAGTAGGTAATCACC***  ***MaMYB4-pro-R: CAATGGCTATCTCACTAAGCTCC***  ***MaACS1-pro-F: GACGATGGCACCGAAAACCAAG***  ***MaACS1-pro-R: GTGACCCGTTATCTCAGGTACG***  ***MaXTH5-pro-F: CTACCATTCTCCCTCAATTGGTTTAT***  ***MaXTH5-pro-R: GGCGTTCTTTCTTTCTTGTGCTC***  ***MaPG3-pro-F: AAGGCCAACATTGAGCTCAGAATG***  ***MaPG3-pro-R: CGTTATCAGCTATGATCTCCTCAG***  ***MaEXPA15-pro-F: GAAGAACTTTGGTCAGTAAGAATCG***  ***MaEXPA15-pro-R: TGGGACAATGAGTGTTGGCCA*** | |  |
| **Dual-luciferase expression assay** | ***MaMYB4/MaMYB4^S160A^-62SK-F: ggccgctctagaactagtggatccATGGGGAGGTCCCCGTG***  ***MaMYB4/MaMYB4^S160A^-62SK-R: atcgataagcttgatatcgaattcTGGGTTTCTGTAGTCCAGCA***  ***MaMPK14-62SK-F: ggccgctctagaactagtggatccATGGCTATGCTGGTTGATCCTC***  ***MaMPK14-62SK-R: atcgataagcttgatatcgaattcGTTAGCAGAAGCTTCTGGATGATA***  ***MaMYB4-pro-0800-F: cactatagggcgaattgggtaccCTCTGTTAATATAGTAGGTAATCACC***  ***MaMYB4-pro-0800-R: tttatgtttttggcgtcttccatCAATGGCTATCTCACTAAGCTCC***  ***MaACS1-pro-0800-F: cactatagggcgaattgggtaccGACGATGGCACCGAAAACCAAG***  ***MaACS1-pro-0800-R: tttatgtttttggcgtcttccatGTGACCCGTTATCTCAGGTACG***  ***MaXTH5-pro-0800-F: cactatagggcgaattgggtaccCTACCATTCTCCCTCAATTGGTTTAT***  ***MaXTH5-pro-0800-R: tttatgtttttggcgtcttccatGGCGTTCTTTCTTTCTTGTGCTC***  ***MaPG3-pro-0800-F: cactatagggcgaattgggtaccAAGGCCAACATTGAGCTCAGAATG***  ***MaPG3-pro-0800-R: tttatgtttttggcgtcttccatCGTTATCAGCTATGATCTCCTCAG***  ***MaEXPA15-pro-0800-F: cactatagggcgaattgggtaccGAAGAACTTTGGTCAGTAAGAATCG***  ***MaEXPA15-pro-0800-R: tttatgtttttggcgtcttccatTGGGACAATGAGTGTTGGCCA*** | | ***Bam*H Ⅰ**  ***Eco*R Ⅰ**  ***Bam*H Ⅰ**  ***Eco*R Ⅰ**  ***kpn* Ⅰ**  ***Nco* Ⅰ**  ***kpn* Ⅰ**  ***Nco* Ⅰ**  ***kpn* Ⅰ**  ***Nco* Ⅰ**  ***kpn* Ⅰ**  ***Nco* Ⅰ**  ***kpn* Ⅰ**  ***Nco* Ⅰ** |
| **Co-IP assay and *In vivo* ubiquitination assay** | ***MaMPK14-His-F: ttctgcccaaattcgcgaccggtATGGCTATGCTGGTTGATCCTC***  ***MaMPK14-His-R: tgatggtgatggtgatgcccgggGTTAGCAGAAGCTTCTGGATGATA***  ***MaMYB4-GFP-F: tattctgcccaaattcgcgaccggtATGGGGAGGTCCCCGTG***  ***MaMYB4-GFP-R: aaagttcttctcctttgctagtcatTGGGTTTCTGTAGTCCAGCA*** | | ***Age* Ⅰ**  ***Xma* I**  ***Age* Ⅰ**  ***Age* Ⅰ** |
| **Firefly luciferase complementation imaging assay** | ***MaMYB4/MaMYB4^S160A^-cLuc-F: acgcgtcccggggcggtaccATGGGGAGGTCCCCGTG***  ***MaMYB4/MaMYB4^S160A^-cLuc-R:*** ***agctctgcaggtcgacCTATGGGTTTCTGTAGTCCAGCA***  ***MaMPK14-nLuc-F: cgggggacgagctcggtaccATGGCTATGCTGGTTGATCCTC***  ***MaMPK14-nLuc-R: acgagatctggtcgacGTTAGCAGAAGCTTCTGGATGATA*** | | ***Kpn* I**  ***Sal* I**  ***Kpn* I**  ***Sal* I** |
| **GST pull down, *in vitro* phosphorylation assay and EMSA assay** | ***MaMYB4/MaMYB4^S160A^-GST-F: ggttccgcgtggatccATGGGGAGGTCCCCGTG***  ***MaMYB4/MaMYB4^S160A^-GST-R: agtcacgatgcggccgcCTATGGGTTTCTGTAGTCCAGCA***  ***MaMPK14-MBP-F: agggaaggatttcagaattcATGGCTATGCTGGTTGATCCTC***  ***MaMPK14-MBP-R: aggtcgactctagaggatccTCAGTTAGCAGAAGCTTCTGGATGATA***  ***MaACS1-probe-F: CCAAAAACAGGGTAGGTGGATGGCTTACATGCTCACCTAATAATTTATTT***  ***MaACS1-probe-R: AAATAAATTATTAGGTGAGCATGTAAGCCATCCACCTACCCTGTTTTTGG*** | | ***Bam*H Ⅰ**  ***Not* Ⅰ**  ***Eco*R Ⅰ**  ***Bam*H Ⅰ** |
| **Banana pulp transient overexpression** | ***MaMPK14-pCXUN-HA -F: ATGGCTATGCTGGTTGATCCTC***  ***MaMPK14-pCXUN-HA -R: TCAGTTAGCAGAAGCTTCTGGATGATA***  ***MaMYB4/MaMYB4^S160A^-pCXUN-HA -F: ATGGGGAGGTCCCCGTG***  ***MaMYB4/MaMYB4^S160A^-pCXUN-HA -R: CTATGGGTTTCTGTAGTCCAGCA*** | | ***Xcm* I**  ***Xcm* I**  ***Xcm* I**  ***Xcm* I** |

**Text S1.** Nucleotide sequences of promoters sequenced for *MaACS1*. YYYACCWAMYW were marked with red boxes. EMSA assay probes were marked with transparent boxes. Translation start site (ATG) was shown in yellow box. 5’ UTR were marked with blue.

***MaACS1* (*Ma04_g35640*)**

GACGATGGCACCGAAAACCAAGAAAGAAATGGGGATGTCCAATCTAAAACGTTGCCGACATCTTAATCGTGCCTGAGATCATATATTACCTACAAACACACGTTATTTATTCTGGCTTTCAGTGGAATAATTACTCTTTACTTTCTTTGATGGATTGCTGGAAATGGATTGTTTGGATTGCGAACGAGAATATAAGAACAACATTCTACTTCAAATATTAATTTCACTTTTGATTCTCGGAAACTACACCCCATAAATTTGATCTTCGAAGCCTAAGAATTAAATCTAAATAATGGTAGAAGCATAATTTATTTTCTACCAAAAACAGGGTAGGTGGATGGCTTACATGCTCACCTAATAATTTATTTAAGTGGGACCCATGAATAGGCCACGTTACATTCAAAATTTGATGATTAGTGTACCATGATGAGCTGTGTAATGCGTGTCTTGATTGCGACTTCCCGTAGACCCCATCGATATTAGATTGAGACGGAAATAACGTCGGTCGGTAGACAACATAGTACGTAGATCATGATAGAACAATTCGAAATAATAAGACATACCAAAAAATTTGGTATACGACTCGATATATAAAAGTTAAAGTTATTTCTTTCATCTCTATATATATATACACAATTTGTAAGTTTCCTAAAAGATATTAGATTTTGGGTCAACTATAATTGCCTTCCAACAAAGTAGAATGCCTCTATCAGAATCTCGCGCCTACAAACAACACCAATCCTAATCATTCACATGATCTTAACGTTAATGAAGTAGCAAATTCCAAATCACATTTAAATAGCCAGTCATTTCAAACATGAAGATCATGCTTTTATTGTTTCTTTTTTGGTAGAAGAGTGAATGAGATCCACATTTTCCTTAAGAGGATGCATATAAGCCAATTAAATTAACATCCATATGTATAAAATCCAATTAGGCAAAGGCAAATCGAAGTAGTCAGATACGTTTCTATCTGATCCGATTAATATTCTCTTTTACGATTGATAAAGAATACATGCTAATTGATATGGGAGGCAATTCCCGACGTATCATACTCATCCGATCGATACGTAATATCGTTAGGTCAGTGATGAAGATGTTAATTTGTCTCACGAGATCGAAATATATAATTAGAAAAGTTGACATGACAAGTCAGTTATTCTATAACCATAGTTTTGATTTCATGACTCATCGTGACATTTATTATCGTCTTAATTTAATTATCATATATTATAATATAAAAGGTGTTCTAGTGGGAGTTTTGACTTAATACATTATTTAATTTTAATGCTTGATTGAATTCTTCAAACTTTTACTAACCTAAATATTGACTAAATAAGTATGCTTCGACAAGATTTTTTCTACGTGATTAAATCTTTGACCTCTTAAACACCTCAACTTGAGTCAAACTCGGAAACTCGAAAGTGGACCTCCGTCAGACTCCTTTAACATCTGTTAAACAACTTGAATCTAATTTGACACTAATCAAAATCAGACTGCATTGAGCCTAAATGGATCTAAATATTAAGAAAACCATTGTTGAATTTTCCTTCTTTCGCAAACAGCATGTCATCGATGAGATTAAGGTTTACAAAGAGCGGCGCACAATTTTGTTTTGGGATAAATAATTCTGTGCTTACAATATAGAAGAGTTCGAGTCGAAAGCGACTCCCGAGTTCGGAACACGTCATTGTTGCCGCCAACACTGAAGCTTCCTATTTGGCGTCACCTGTCGATGTTACGGCGCATCCATCGCCAATCACGTCCATGATTTACACGCTGCCGGATCGACTCGGTTTTCATGTCCTTCTTTTCCAGCCTGAAGTCCTCTTTGTTGACCTCTTTGGATGTTTGAATGGTCTCGGGATTTGCCTATTAATGGTCATCGGAATCGACTCTTGCAAACTGCAGCAGCTGCTTCTCCTTCTTCTTCTCTGCTCGCTTCAGCCTTTTCCGGTACGTACCTGAGATAACGGGTCACATG
